# Reversible Addition-Fragmentation Chain Transfer Aqueous Dispersion Polymerization of 4-Hydroxybutyl Acrylate Produces Highly Thermoresponsive Diblock Copolymer Nano-Objects

**DOI:** 10.1021/acs.macromol.1c02431

**Published:** 2022-01-19

**Authors:** Juliana M. Cumming, Oliver J. Deane, Steven P. Armes

**Affiliations:** Dainton Building, Department of Chemistry, University of Sheffield, Brook Hill, Sheffield, South Yorkshire S3 7HF, UK

## Abstract

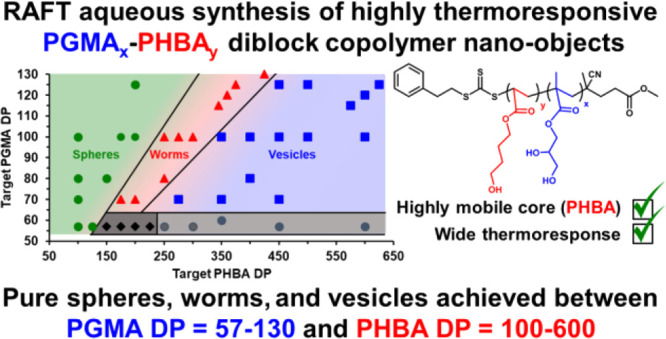

The reversible addition-fragmentation chain transfer (RAFT) aqueous dispersion polymerization of 2-hydroxypropyl methacrylate (HPMA) using a poly(glycerol monomethacrylate) (PGMA) precursor is an important prototypical example of polymerization-induced self-assembly. 4-Hydroxybutyl acrylate (HBA) is a structural isomer of HPMA, but the former monomer exhibits appreciably higher aqueous solubility. For the two corresponding homopolymers, PHBA is more weakly hydrophobic than PHPMA. Moreover, PHBA has a significantly lower glass transition temperature (*T*_g_) so it exhibits much higher chain mobility than PHPMA at around ambient temperature. In view of these striking differences, we have examined the RAFT aqueous dispersion polymerization of HBA using a PGMA precursor with the aim of producing a series of PGMA_57–300_-PHBA_100–1580_ diblock copolymer nano-objects by systematic variation of the mean degree of polymerization of each block. A pseudo-phase diagram is constructed using transmission electron microscopy to assign the copolymer morphology after employing glutaraldehyde to cross-link the PHBA chains and hence prevent film formation during grid preparation. The thermoresponsive character of the as-synthesized linear nano-objects is explored using dynamic light scattering and temperature-dependent rheological measurements. Comparison with the analogous PGMA_*x*_-PHPMA_*y*_ formulation is made where appropriate. In particular, we demonstrate that replacing the structure-directing PHPMA block with PHBA leads to significantly greater thermoresponsive behavior over a much wider range of diblock copolymer compositions. Given that PGMA-PHPMA worm gels can induce stasis in human stem cells (see Canton *et al.*, *ACS Central Science*, 2016, *2*, 65–74), our findings are likely to have implications for the design of next-generation PGMA-PHBA worm gels for cell biology applications.

## Introduction

Polymerization-induced self-assembly (PISA) offers a highly effective and versatile route to bespoke block copolymer nano-objects.^1−10^ Typically, PISA involves growing an insoluble block from one end of a soluble block in a suitable selective solvent: self-assembly occurs *in situ* on reaching a certain critical degree of polymerization, which eventually leads to the production of sterically stabilized nanoparticles. When such syntheses are conducted using reversible addition-fragmentation chain transfer (RAFT) polymerization, PISA can be used to design a wide range of functional block copolymer nanoparticles directly in aqueous media.^11−17^

Depending on the solubility of the vinyl monomer used to produce the hydrophobic block, this can be achieved by either RAFT aqueous emulsion polymerization (for water-immiscible monomers)^5,8,18−36^ or RAFT aqueous dispersion polymerization (for water-miscible monomers).^15,37−59^ However, only the latter approach yields shape-shifting thermoresponsive block copolymer nano-objects. This is because the structure-directing block is only weakly hydrophobic in this case; hence, subtle changes in its (partial) degree of hydration on either heating or cooling can induce morphological transitions.^12,46,51,52,60−62^ There are many examples of RAFT aqueous dispersion polymerization reported in the literature.^12,13,15,37,39,42,43,47,49−51,57−59,63−75^ However, the prototypical—and certainly most widely explored—formulation is based on the RAFT aqueous dispersion polymerization of 2-hydroxypropyl methacrylate (HPMA). Various water-soluble polymers have been used as a precursor block for such formulations, including poly(glycerol monomethacrylate) (PGMA), poly(2-(methacryloyloxy)ethyl phosphorylcholine) (PMPC), poly(ethylene glycol) (PEG), and poly(2-hydroxypropyl methacrylamide) (PHPMAC).^19,46,65,67,76−80^

Blanazs *et al.* reported the first example of thermoresponsive block copolymer nano-objects prepared *via* PISA.^77^ Notably, cooling a 10% w/w aqueous dispersion of PGMA_54_-PHPMA_140_ worms from 20 to 4 °C led to the formation of spheres. Moreover, this morphological transition proved to be reversible and was accompanied by *in situ* degelation, which enabled convenient sterilization of the initial worm gel *via* cold ultrafiltration.^77^ With appropriate purification, such worm gels are sufficiently biocompatible to enable cell biology studies to be conducted with various cell lines.^81−85^ Similarly, PGMA_58_-PHPMA_250_ vesicles are also thermoresponsive and can be converted into spheres at sub-ambient temperature.^86^ Such behavior can be used to trigger the release of a nanoparticle payload within the original vesicles.^86−91^ More recently, Ratcliffe *et al.* reported that a single PHPMAC_41_-PHPMA_180_ diblock copolymer can form spheres, worms, or vesicles in aqueous solution depending solely on the temperature.^46^ On the other hand, Warren and co-workers reported that thermoresponsive behavior is no longer observed if the mean degree of polymerization of PHPMA is too high.^76^ This latter study begs the following question: is there an alternative water-miscible monomer to HPMA that can confer greater thermoresponsive character on block copolymer nano-objects prepared *via* RAFT aqueous dispersion polymerization?

**Scheme 1:**
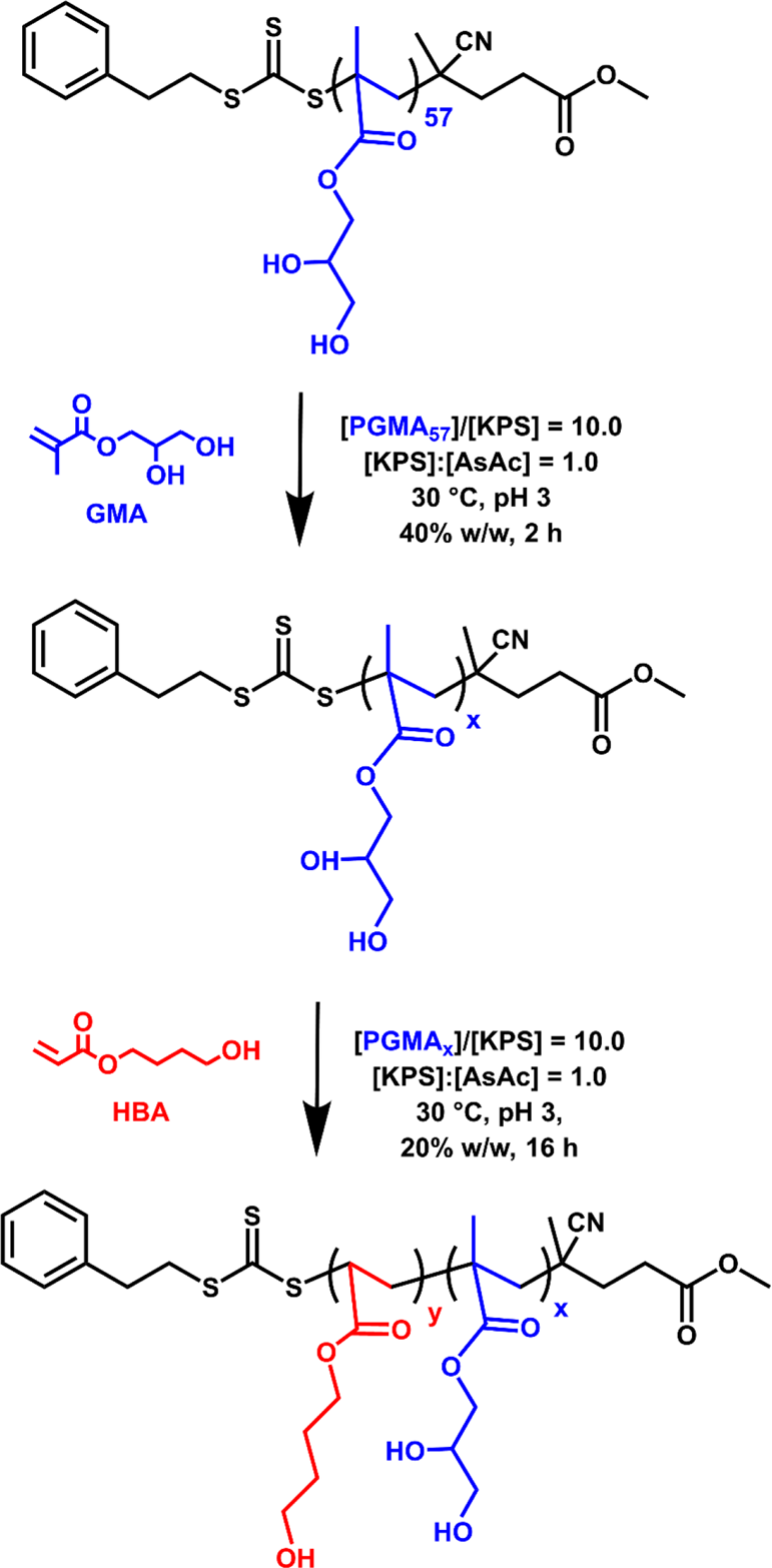
Reaction Scheme for the RAFT Aqueous Solution Polymerization of GMA Using a PGMA_57_ Precursor to Produce a Longer PGMA_*x*_ (*x* = 70–300) Stabilizer Block, Followed Immediately by the RAFT Aqueous Dispersion Polymerization of HBA at 30 °C to Obtain a Series of PGMA_*x*_-PHBA_*y*_ Diblock Copolymer Nano-Objects

Herein, we demonstrate that 4-hydroxybutyl acrylate (HBA) offers a very useful alternative to HPMA in this context. Although HBA and HPMA are structural isomers, HBA is miscible with water in all proportions, whereas the aqueous solubility of HPMA is only 13% w/w at 20 °C.^92^ This observation immediately suggests that PHBA should be more weakly hydrophobic than PHPMA. Moreover, the much lower glass transition temperature of the former homopolymer should ensure significantly greater chain mobility, with such differences likely to confer greater thermoresponsive character. Indeed, our direct comparison of the aqueous solution behavior exhibited by PEG-PHPMA and PEG-PHBA nano-objects confirms this prediction.^93^ In this prior study, choosing PEG as the steric stabilizer block facilitated variable temperature ^1^H NMR spectroscopy experiments that revealed an unexpected qualitative difference between the thermoresponsive behavior exhibited by these two diblock copolymers. In the present study, we compare the thermoresponsive behavior of a series of PGMA-PHBA diblock copolymer nano-objects with the analogous PGMA*_x_*-PHPMA*_y_* nano-objects (see Scheme 1) with the analogous PGMA*_x_*-PHPMA*_y_* nano-objects. PGMA-PHBA worms are potentially interesting in the context of cell biology applications because the hydroxyl-rich nature of the PGMA stabilizer block can induce stasis in human stem cell colonies immersed within PGMA_55_-PHPMA_135_ worm gels, whereas the same cells continue to proliferate when immersed within a PEG_57_-PHPMA_65_ worm gel of comparable softness.^83,84^

## Results and Discussion

### Synthesis of PGMA_*x*_-PHBA_*y*_ Diblock Copolymer Nano-Objects

The diblock copolymer nano-objects described herein were synthesized starting from a PGMA_57_ precursor prepared using a non-ionic trithiocarbonate-based RAFT agent (methyl 4-cyano-4-(2-phenylethylsulfanylthiocarbonyl)sulfanylpentanoate, Me-PETTC), as reported elsewhere.^94^ This is an important choice of RAFT agent because it avoids the formation of anionic carboxylate end-groups at physiological pH, which can induce either worm-to-sphere or vesicle-to-worm morphological transitions.^95^ However, preliminary experiments performed using PGMA_57_ indicated that this precursor was too short to confer colloidal stabilization when targeting longer PHBA blocks (see later). Moreover, Me-PETTC is insoluble in water so solution polymerizations performed using this RAFT agent are typically performed in non-aqueous media.^94^ Thus, for the convenient preparation of PGMA precursors with relatively high DPs in wholly aqueous media, this PGMA_57_ precursor was initially chain-extended *via* RAFT aqueous solution polymerization of GMA. This step was conducted at 40% w/w solids using a well-known low-temperature redox initiator^96−98^ based on potassium persulfate (KPS) and ascorbic acid (AsAc), see Scheme 1.

**Figure 1:**
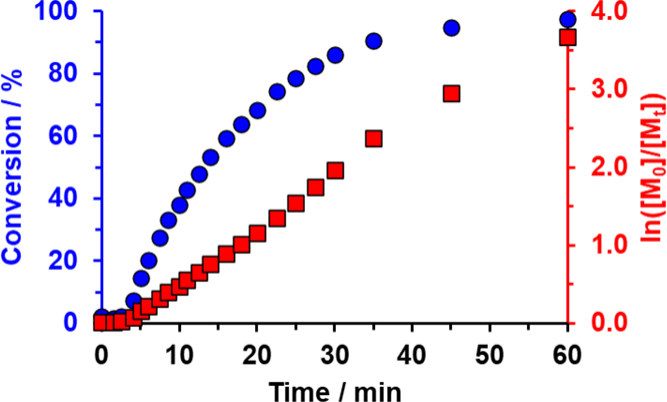
Conversion vs time curve (blue circles) and the corresponding semilogarithmic plot (red squares) obtained for the RAFT aqueous dispersion polymerization of HBA using a KPS/AsAc redox initiator at 30 °C when targeting PGMA_100_-PHBA_650_ diblock copolymer nano-objects at 20% w/w solids at a [PGMA_100_]/[KPS] molar ratio of 10.

**Figure 2:**
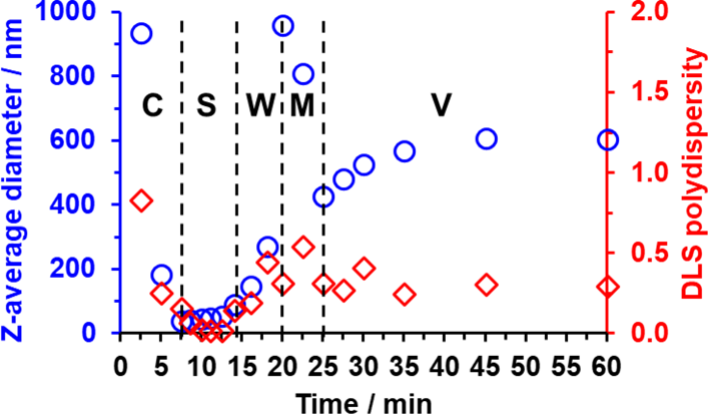
Evolution in *z*-average diameter and polydispersity determined by DLS studies during the synthesis of PGMA_100_-PHBA_650_ diblock copolymer nano-objects *via* RAFT aqueous dispersion polymerization of HBA using a KPS/AsAc redox initiator at 30 °C when targeting 20% w/w solids. Aliquots were extracted periodically over 60 min. [N.B. **C**, **S**, **W**, **M**, and **V** denote chains, spheres, worms, a mixed phase comprising worms and vesicles, and vesicles, respectively].

^1^H NMR studies indicated that more than 99% GMA conversion was achieved within 90 min at 30 °C when targeting an overall PGMA DP of 100. This chain-extended precursor was then used directly (i.e., without isolation or purification) for the subsequent RAFT aqueous dispersion polymerization of HBA at 20% w/w solids when targeting a PHBA DP of 650 at 30 °C (see Scheme 1). A kinetic study was performed for this aqueous PISA formulation by periodic sampling of the reaction mixture (see the Experimental Section in the Supporting Information for further details). ^1^H NMR studies indicated a relatively slow rate of polymerization for the first 4 min (see Figure 1). Thereafter, the rate of HBA polymerization exhibited first-order kinetics with respect to the monomer and more than 99% conversion was achieved within 60 min at 30 °C. The aliquots extracted during this kinetic study were also used to make up 0.1% w/w copolymer dispersions for dynamic light scattering (DLS) studies at 30 °C (Figure 2).

**Figure 3:**
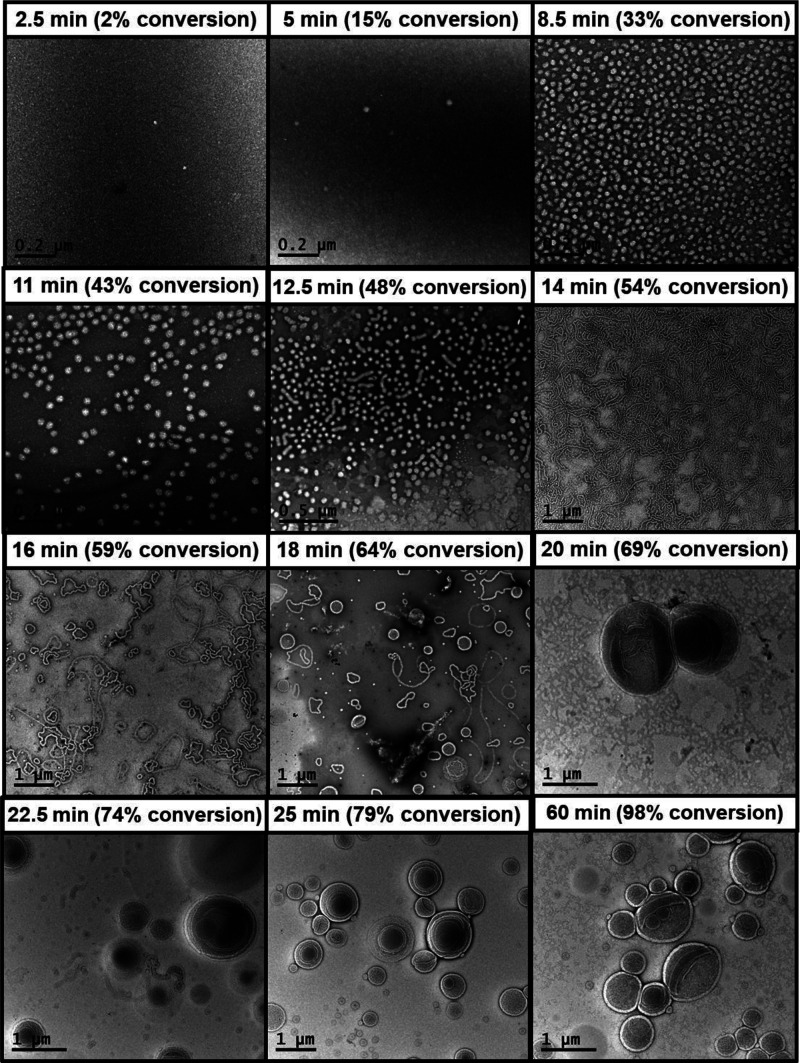
Representative TEM images obtained for aliquots extracted over 60 min during the synthesis of PGMA_100_-PHBA_650_ vesicles *via* RAFT aqueous dispersion polymerization of HBA, illustrating the progressive evolution in the copolymer morphology from spheres to worms to vesicles. In each case, the nano-object morphology was covalently stabilized at 0.1% w/w solids using excess GA cross-linker at 30 °C (to mimic the PISA synthesis conditions).

This technique indicated that relatively small, well-defined spheres with a *z*-average diameter of 36 nm (DLS polydispersity = 0.03) were formed after 7.5 min, see Figure 2. This time point corresponds to around 28% conversion (see Figure 1), thus indicating a critical PHBA DP of approximately 180. Given that PHBA is more weakly hydrophobic than PHPMA and a critical PHPMA DP of 80–90 has been reported for micellar nucleation during the RAFT aqueous dispersion polymerization of HPMA, these observations seem to be physically reasonable.^49,99^ Thus, the upturn observed in the ^1^H NMR-derived kinetic data after 4 min (see Figure 1) most likely corresponds to the onset of polymerization after mild retardation, rather than micellar nucleation. This is because the corresponding critical PHBA DP of 47 seems to be too low to induce microphase separation. It is also notable that no discernible rate enhancement is observed after 7.5 min, which suggests that there is no significant partitioning of the unreacted HBA monomer within the nascent PHBA-core nanoparticles. In contrast, a five-fold increase in the rate of polymerization was observed after micellar nucleation during the RAFT aqueous dispersion polymerization of HPMA.^67^ This marked difference is presumably because HPMA has relatively limited aqueous solubility (13% w/w at 20 °C), whereas HBA is fully miscible with water in all proportions. After nucleation, the *z*-average diameter increased up to 51 nm (DLS polydispersity = 0.02). After 14 min, there was a significant increase in both the apparent *z*-average diameter and polydispersity (87 nm and 0.14, respectively; see Figure 2). The PISA literature suggests that such changes most likely correspond to the formation of short worms.^100^ After 20 min, the apparent *z*-average diameter and DLS polydispersity increased substantially to 957 and 0.32 nm, respectively, which suggests the presence of relatively long, polydisperse worms. Between 20 and 25 min, the *z*-average diameter and DLS polydispersity vary significantly, which suggests that DLS cannot be used to identify the nano-objects present in these aliquots (see Figure 3 and accompanying text for further discussion). A significant reduction in *z*-average diameter is observed after 25 min, which is consistent with the onset of a worm-to-vesicle transition. Thereafter, the DLS polydispersity is reduced to around 0.25–0.35 and the *z*-average diameter rises to 606 nm after 45 min (Figure 2). ^1^H NMR spectroscopy studies confirm that essentially full HBA conversion is achieved within 60 min (Figure 1).

In the PISA literature, transmission electron microscopy (TEM) is routinely used to determine the copolymer morphology.^4,49,78^ However, the *T*_g_ of the PHBA block is below −30 °C,^62,93^ which invariably leads to film formation during TEM grid preparation and thus prevents morphological assignment. Byard *et al.* addressed this technical problem by statistically copolymerizing HBA with diacetone acrylamide (DAAM) to enable the resulting ketone-functionalized nano-objects to be cross-linked with adipic acid dihydrazide (ADH) prior to TEM studies.^51^ This approach enabled high-quality TEM images to be obtained, but introducing the DAAM comonomer reduced the thermoresponsive character of the nano-objects.^101^ To avoid this undesirable limitation, we recently reported the covalent stabilization of PHBA-based nano-objects using glutaraldehyde (GA).^62^ In principle, one GA molecule can react with four hydroxy groups on the PHBA chains to form two stable acetal linkages.^102^ This implies that a GA/HBA molar ratio of 0.25 should be sufficient to avoid film formation during TEM grid preparation. Empirically, we found that excess GA was required to ensure high-quality TEM images.^62^ More specifically, a GA/HBA molar ratio of 1.0 was used to cross-link the various nano-objects produced when targeting PGMA_100_-PHBA_650_ vesicles in the present study (see the Supporting Information for further details). The resulting TEM images shown in Figure 3 are in reasonably good agreement with the DLS data reported in Figure 2. Initially, there is no TEM evidence for the presence of any nano-objects. After 7.5 min, spherical nano-objects with a number-average diameter of 34 ± 5 nm (*z*-average diameter = 36 nm) are observed. At around 12.5 min, these nascent spheres began to undergo fusion to form dimers and trimers, with a pure phase of longer worms being observed after 14 min (TEM mean worm contour length = 406 ± 258 nm, whereas DLS reports a “sphere-equivalent” *z*-average diameter of 87 nm). Toroidal nano-objects, which are rarely reported in the PISA literature, can be identified after 16 min. Interestingly, there was no evidence for the presence of “jellyfish”-type intermediates during this aqueous PISA synthesis. Vesicles were first observed after 20 min, albeit as a mixed phase co-existing with worms. A pure phase comprising oligolamellar vesicles was obtained after 25 min.

For other PISA formulations, vesicles reach a certain limiting diameter and further polymerization merely results in thicker vesicle membranes according to an “inward growth” mechanism.^103,104^ In contrast, the current PISA formulation does not appear to follow such a vesicle growth mechanism because DLS studies suggest that the overall vesicle diameter increases monotonically during the final stages of the HBA polymerization (see Figure 2). However, the oligolamellar morphology may well be a complicating factor and this intriguing aspect clearly warrants further investigation in the future using time-resolved small-angle X-ray scattering (SAXS).^49^

**Figure 4:**
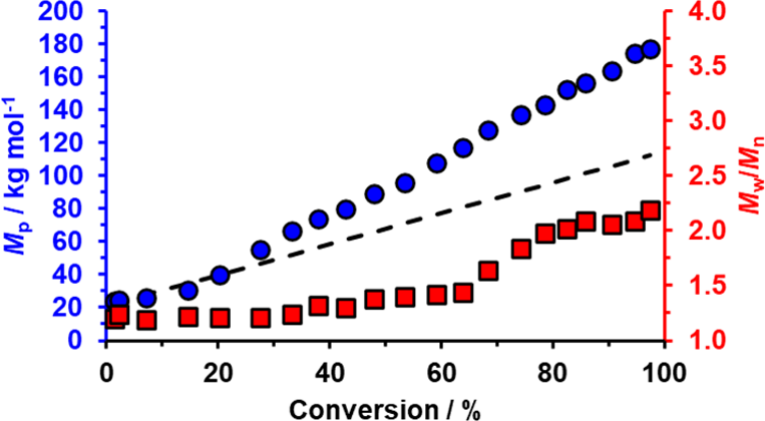
Evolution in diblock copolymer *M*_p_ and *M*_w_/*M*_n_ with HBA conversion determined by DMF GPC (expressed against a series of poly(methyl methacrylate) calibration standards) during the RAFT aqueous dispersion polymerization of HBA using a KPS/AsAc redox initiator at 30 °C when targeting PGMA_100_-PHBA_650_ vesicles at 20% w/w solids using a [PGMA_100_]/[KPS] molar ratio of 10. The dashed black line corresponds to the theoretical molecular weight for the PGMA_100_-PHBA_*x*_ diblock copolymer chains.

DMF gel permeation chromatography (GPC) studies indicate a reasonably linear evolution in copolymer *M*_p_ with HBA monomer conversion (Figure 4). The non-zero *y*-intercept corresponds to an apparent *M*_p_ of 20.3 kg mol^–1^ for the PGMA_100_ precursor (as expressed relative to PMMA calibration standards). Moreover, comparison of GPC curves recorded for the diblock copolymers with that obtained for the precursor indicates that relatively efficient chain extension (>90%) is achieved, see Figure S1. However, MWDs become significantly broader (*M*_w_/*M*_n_ > 1.30) above 50% HBA conversion. Inspection of the corresponding GPC curves (Figure S1) confirms the progressive development of a high molecular weight shoulder, which results in a relatively high final dispersity (*M*_w_/*M*_n_ = 2.19). Notably, GPC curves recorded using a UV detector tuned to the wavelength of the organosulfur RAFT agent (λ = 302 nm) overlay with those obtained using the refractive index detector (e.g., see Figure S2). This suggests that significant chain transfer to polymer occurs during these syntheses rather than premature hydrolysis of trithiocarbonate end-groups, which would inevitably result in loss of RAFT control.^105,106^ This side-reaction is well known for acrylic monomers such as HBA, especially when targeting relatively high DPs and even at reaction temperatures as low as 30 °C.^107^

Overall, these data are typical for a pseudo-living radical polymerization when targeting a relatively high DP for an acrylic block.^62,93,106,108^ Fortunately, the PISA literature suggests that even relatively polydisperse diblock copolymer chains can self-assemble to form relatively well-defined nano-objects.^67,107,109−112^

### Construction of a Pseudo-Phase Diagram for PGMA_*x*_-PHBA_*y*_ Nano-Objects

Blanazs *et al.* were the first to demonstrate the critical role of the steric stabilizer DP for PISA formulations.^65^ Three pseudo-phase diagrams were constructed for PGMA_*x*_-PHPMA_*y*_ nano-objects by systematically varying the PHPMA DP and copolymer concentration using PGMA precursors with mean DPs (*x*) of 47, 78, or 112. According to the PISA literature, spheres undergo stochastic 1D sphere–sphere fusion to form worms.^113^ However, Blanazs *et al.* found that pure worms or vesicles could not be accessed when using the PGMA_112_ precursor—instead, the phase diagram was dominated by kinetically-trapped spheres.^65^ This is because the relatively long hydrophilic block confers highly effective steric stabilization and hence prevents sphere–sphere fusion, which is the critical first step in the evolution of the copolymer morphology.^68^ In contrast, the PGMA_78_-PHPMA_*y*_ formulation provided access to spheres, worms or vesicles depending on the copolymer concentration.^65^ Further reduction in the stabilizer block DP (i.e., PGMA_47_) eliminated this concentration dependence while still providing access to all three copolymer morphologies.^65^ Given that HBA is a structural isomer of HPMA, similar behavior was anticipated for the PGMA_*x*_-PHBA_*y*_ system reported herein.

**Figure 5:**
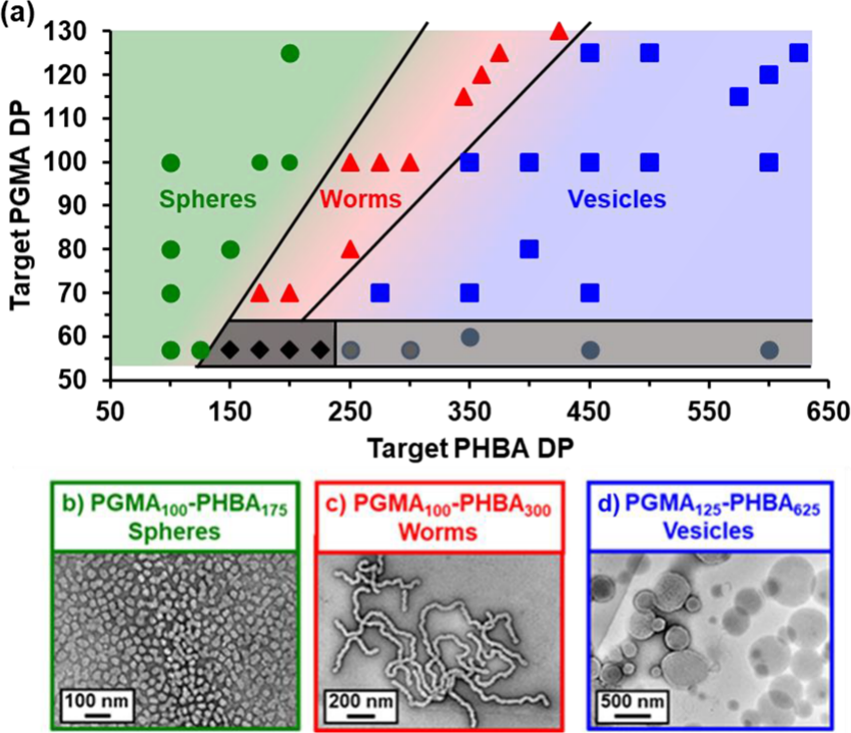
(a) Master pseudo-phase diagram constructed for a series of PGMA_*x*_-PHBA_*y*_ nano-objects after covalent stabilization at 20 °C using GA as a cross-linker. All syntheses involved the RAFT aqueous dispersion polymerization of HBA at 20% w/w solids at 30 °C. Each point represents the copolymer morphology assigned on the basis of DLS and TEM studies. Green circles indicate spheres, red triangles indicate worms, blue squares indicate vesicles, black filled diamonds indicate mixed sphere/worms, and gray circles indicate macroscopic precipitation. Representative TEM images obtained for (b) GA-cross-linked PGMA_100_-PHBA_175_ spheres, (c) GA-cross-linked PGMA_100_-PHBA_300_ worms, and (d) GA-cross-linked PGMA_125_-PHBA_625_ vesicles.

Accordingly, a series of PGMA_*x*_-PHBA_*y*_ nano-objects were prepared at 20% w/w solids using the KPS/AsAc redox initiator at 30 °C (Scheme 1) to identify the maximum PGMA DP that still provided access to the full range of copolymer morphologies. Initially, a series of nine PGMA precursors with target DPs of 57, 60, 70, 80, 100, 115, 120, 125, and 130 were prepared. DMF GPC analysis indicated that relatively narrow MWDs were obtained in each case and confirmed a linear increase in *M*_n_ with target PGMA DP (*M*_n_ = 14.3–32.6 kg mol^–1^ and *M*_w_/*M*_n_ = 1.21–1.32; Table S1). These precursors were then chain-extended in turn *via* RAFT aqueous dispersion polymerization of HBA while targeting PHBA DPs ranging from 100 to 625. DLS and TEM studies were used to assign the various copolymer morphologies and hence construct the pseudo-phase diagram shown in Figure 5.

When using PGMA_57_ or PGMA_60_, only spherical nano-objects could be obtained if PHBA DPs below 150 were targeted (see Figure 5a). Increasing the target PHBA DP using either of these two relatively short precursors initially resulted in mixed phases (PHBA DP = 150–225; see Figure 5a) but ultimately only led to macroscopic precipitation (PHBA DP > 250). Clearly, neither PGMA_57_ nor PGMA_60_ confers sufficient steric stabilization when targeting higher PHBA DPs.

In contrast, a PGMA_70_ precursor enabled the synthesis of colloidally stable spheres (PHBA DP = 100), worms (PHBA DP = 150 or 200), or vesicles (PHBA DP > 275). Presumably, this is close to the minimum PGMA DP required to ensure colloidal stability. Moreover, as discussed above, the PGMA_100_ precursor enabled the synthesis of pure spheres (for PHBA DPs of between 100 and 200), worms (PHBA DPs = 250–300), and vesicles (PHBA DPs = 350–600), which is in striking contrast to the observations made by Blanazs *et al.* for PGMA_112_-PHPMA_*x*_ formulations.^65^ This suggests that the much greater chain mobility of the weakly hydrophobic PHBA block (relative to that of the PHPMA block) makes a decisive difference in determining the behavior of their respective aqueous PISA formulations.

In principle, pseudo-phase diagrams such as that shown in Figure 5 can be used to predict copolymer morphologies. Hence the same PHBA/PGMA molar ratios corresponding to pure worms (PHBA/PGMA = 2.50–3.25) and pure vesicles (PHBA/PGMA = 5.0) were targeted when employing longer PGMA precursors.^76^ Gratifyingly, this rational approach enabled rapid identification of the diblock compositions required to produce pure worms and vesicles when chain-extending the PGMA_115–130_ precursors (see Figure 5 for the corresponding TEM images and the pseudo-phase diagram shown in Figure S3).

**Figure 6:**
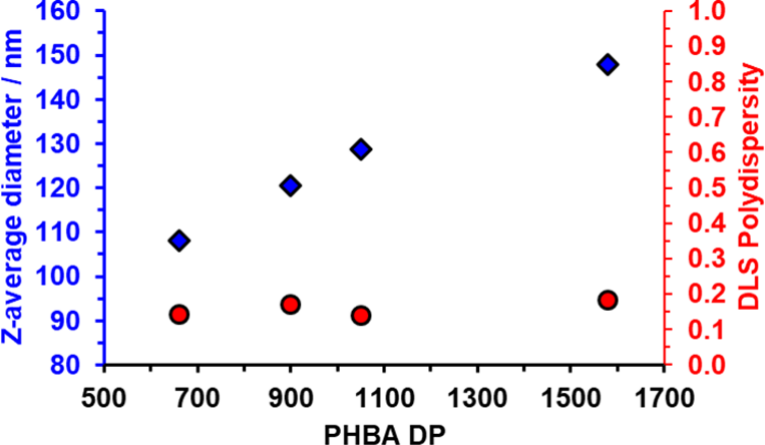
Variation in *z*-average diameter and polydispersity determined *via* DLS studies of a series of kinetically-trapped PGMA_300_-PHBA_*x*_ spheres prepared at 30 °C when targeting a PHBA DP (*x*) ranging from 660 to 1580 at 20% w/w solids. [N.B. Targeting even higher PHBA DPs merely resulted in substantially incomplete HBA conversions (<90%) under the same conditions].

As the upper limit PGMA DP for kinetically-trapped spheres had not yet been identified for the RAFT aqueous dispersion polymerization of HBA, a PGMA_140_ precursor was prepared and subsequently chain-extended while targeting a PHBA DP of 420. According to Figure 5a, this HBA/GMA molar ratio of 3.0 should result in the formation of worms. However, DLS studies indicated that only kinetically-trapped spheres (*z*-average diameter = 92 nm; DLS polydispersity = 0.04) were obtained and this morphological assignment was subsequently confirmed by TEM studies (see Figures S3 and S4). Similarly, only kinetically trapped spheres were obtained when using either PGMA_250_ or PGMA_300_ precursors for the RAFT aqueous dispersion polymerization of HBA. As expected, increasing the PHBA DP simply resulted in a monotonic increase in *z*-average diameter for such aqueous PISA formulations (see Figure 6 and Figure S4). Finally, targeting PGMA_150_-PHBA_700_ and PGMA_200_-PHBA_700_ nano-objects led to the formation of a mixed phase comprising spheres and worms (see Figure S4). In summary, the upper limit PGMA DP that still provides access to pure worms and vesicles appears to lie between 130 and 140. Clearly, this is significantly greater than that observed for the RAFT aqueous dispersion polymerization of HPMA.^65^

### Thermoresponsive Behavior of PGMA_*x*_-PHBA_*y*_ Nano-Objects

**Figure 7:**
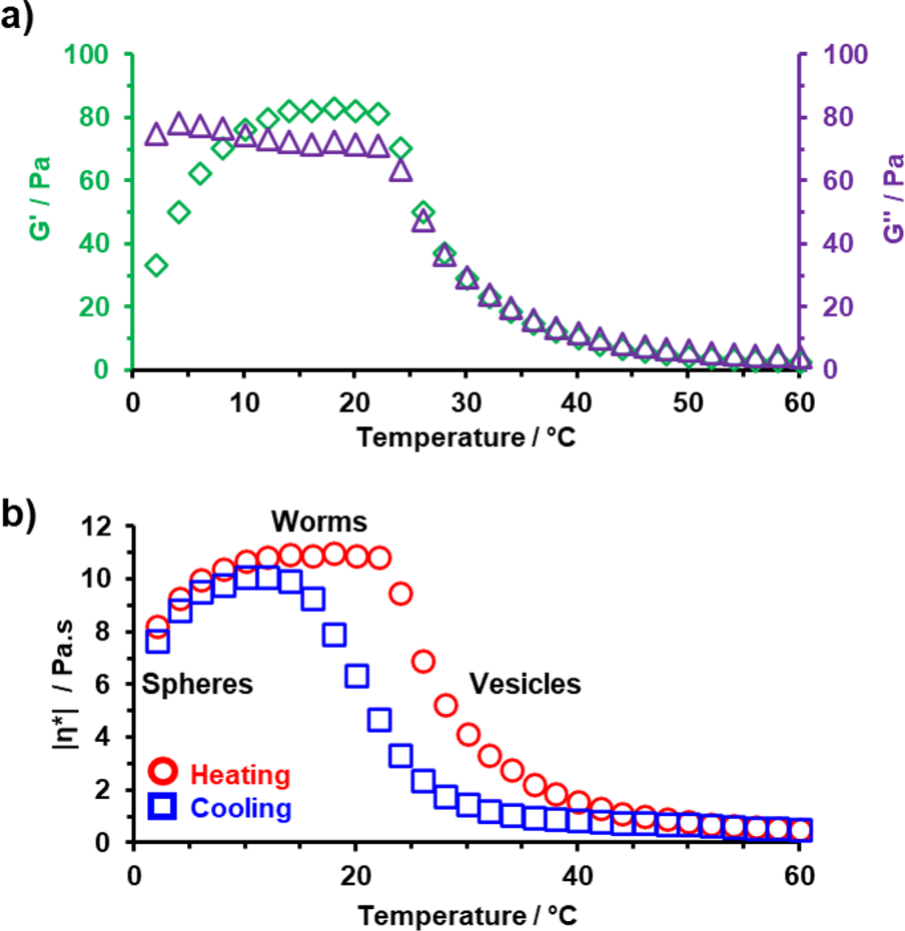
Temperature-dependent rheology studies of a 10% w/w aqueous dispersion of linear PGMA_100_-PHBA_325_ nano-objects recorded at an applied strain of 1.0% and an angular frequency of 1.0 rad s^–1^: (a) storage (*G*′; green diamonds) and loss (*G*″; purple triangles) moduli; (b) complex viscosity (heating ramp = red data; cooling ramp = blue data). For each measurement, 2.0 min was allowed for thermal equilibration.

Recently, we reported the remarkable thermoreversible behavior of PHBA-based nano-objects that undergo an evolution in copolymer morphology from spheres to worms to vesicles to lamellae when increasing the dispersion temperature from 1 to 70 °C.^62^ Variable temperature ^1^H NMR studies indicated that the PHBA block became more hydrated on heating, indicating a uniform plasticization mechanism.^93^ In these two prior reports, the steric stabilizer blocks comprised either poly(2-(*N*-acryloyloxy)ethyl pyrrolidone)^62^ or poly(ethylene glycol).^93^ In principle, the PGMA_*x*_-PHBA_*y*_ nano-objects discussed above should exhibit comparable thermoresponsive behavior. To explore this hypothesis, temperature-dependent rheological studies were conducted on a 10% w/w aqueous dispersion of linear PGMA_100_-PHBA_325_ nano-objects between 2 and 60 °C (Figure 7). At 2 °C, the storage modulus (*G*′) is significantly lower than the loss modulus (*G*″), which indicates a free-flowing fluid (as confirmed by visual inspection of the dispersion). On warming to 10 °C, *G*′ just exceeds *G*″, indicating the formation of a physical gel owing to the generation of highly anisotropic interacting worms, which form a 3D network *via* multiple inter-worm contacts.^61^ This temperature is designated as the critical gelation temperature (CGT). At 30 °C, *G*″ exceeds *G*′ and the concomitant degelation corresponds to a worm-to-vesicle transition. This is consistent with the copolymer morphology assignment made based on the variable temperature DLS data and visual inspection, which confirmed a transition from a free-standing gel (which forms above 10 °C) to a free-flowing turbid dispersion above 30 °C, see Figure S5.

Thermoreversibility was then examined by determining the complex viscosity (|η*|) of this 10% w/w aqueous copolymer dispersion during the same thermal cycle (Figure 7b). Clearly, the sphere-to-worm and worm-to-sphere transitions are more or less reversible, although some hysteresis was observed for the vesicle-to-worm transition during the cooling run. In contrast, the other HBA-based diblock copolymer systems previously reported by our group exhibited essentially no hysteresis during their sphere-to-worm and worm-to-vesicle transitions.^51,62^ In principle, the hysteresis observed in the present study may be related to the stronger hydrogen bonding interactions afforded by the *cis*-diol groups on the PGMA chains compared to the non-hydroxyl-functional stabilizer blocks that have been previously reported.^51,62,93^ However, further studies are required to corroborate this hypothesis.

Prior PHBA-based worm formulations exhibited *G*′ values ranging between 20 and 100 Pa, with higher *G*′ values being reported for more concentrated aqueous dispersions [e.g., *G*′ ∼30 Pa for a 10% w/w PNAEP_85_-PHBA_295_ worm gel and *G*′ ∼100 Pa for a 20% w/w PDMAC_56_-P(0.80 HBA-*stat*-0.20 DAAM)_264_ worm gel].^51,62^ The ability to systematically tune the storage modulus of a worm gel for a given diblock copolymer system is likely to be useful for potential biomedical applications. For example, relatively soft worm gels with *G*′ values of 10–50 Pa can induce stasis in human pluripotent stem cells (and possibly also human embryos).^84^

**Figure 8:**
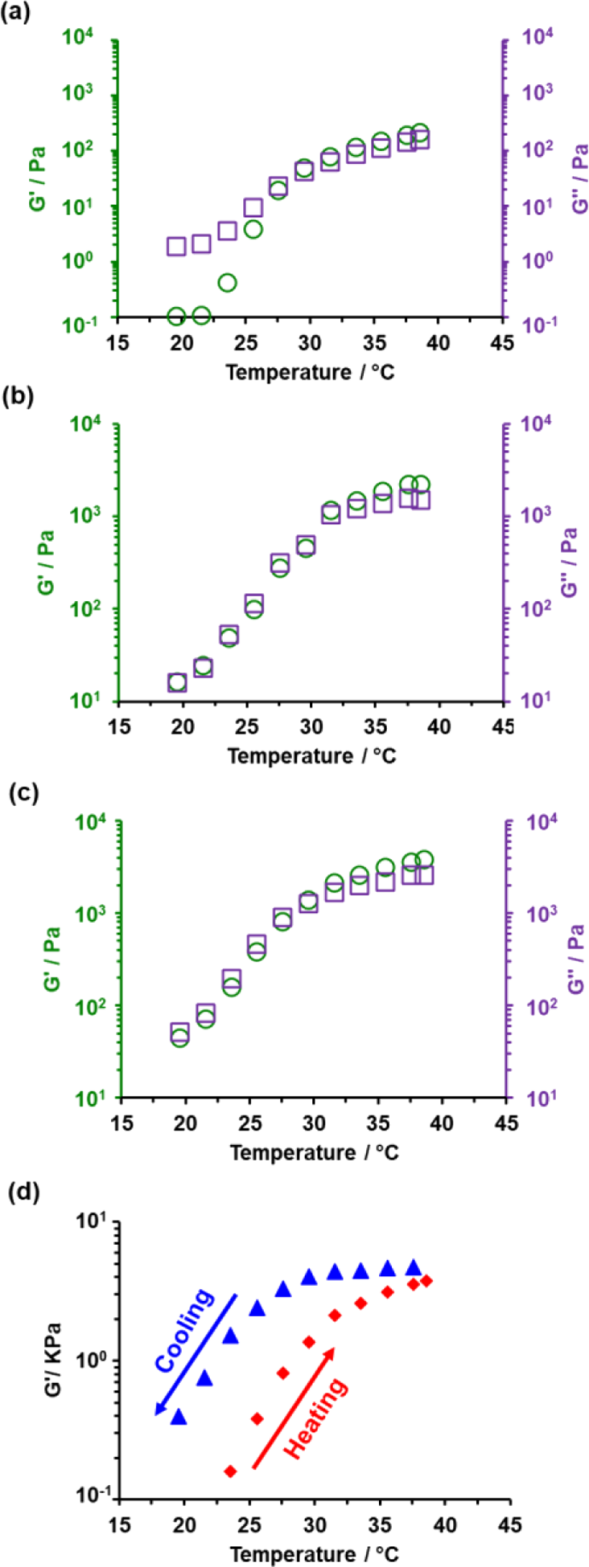
Temperature-dependent rheology studies of 20% w/w aqueous dispersions recorded at an applied strain of 1.0% and an angular frequency of 1.0 rad s^–1^. Storage (*G*′ = green circles) and loss (*G*″ = purple squares) moduli observed during a heating ramp from 20 to 37 °C for the following linear nano-objects: (a) PGMA_70_-PHBA_150_, (b) PGMA_100_-PHBA_210_, and (c) PGMA_130_-PHBA_270_. (d) *G*′ values observed for PGMA_130_-PHBA_270_ nano-objects during heating (red diamonds) and cooling (blue triangles) cycles between 20 and 37 °C. For each measurement, 2 min was allowed for thermal equilibration.

In the present study, three 20% w/w aqueous dispersions of PGMA_*x*_-PHBA_*y*_ nano-objects (where *x* = 70, 100, or 130 and *y* = 150, 210, or 270) were prepared such that the HBA/GMA molar ratio remained approximately 2.1. Visual inspection indicated that each dispersion was viscous but free-flowing. DLS studies indicated relatively high polydispersities in each case (DLS polydispersity > 0.15). These observations are consistent with a mixed phase comprising spheres and worms, which is consistent with the copolymer morphology predicted by the pseudo-phase diagram shown in Figure 5. Rheological studies were performed during a 38–15–38 °C thermal cycle. Initial cooling to 15 °C was required to thermally “reset” the dispersion and hence ensure reproducible data.^114^ It is perhaps worth noting that this precaution was not required when conducting rheology studies on PHBA-based nano-objects prepared with alternative stabilizer blocks, which suggests that the PGMA stabilizer chains may contribute to this thermal history problem. The *G*′ (red circles) and *G*″ (blue squares) data recorded during the subsequent heating run are shown in Figure 8a–c.

These studies indicate that physical gelation (*G*′ > *G*″) occurred at a CGT of approximately 30–32 °C for each of the three aqueous dispersions. Moreover, increasing the PHBA DP (while maintaining an approximately constant HBA/GMA molar ratio of 2.1) resulted in progressively stronger worm gels being formed at 37 °C (Figures 8a–c). The *G*′ values at 37 °C were 220, 2250, and 3790 Pa for the PGMA_70_-PHBA_150_, PGMA_100_-PHBA_210_, and PGMA_130_-PHBA_270_ worm gels, respectively (see Figure S6 for the corresponding TEM images). Importantly, these studies were performed at 20% w/w solids, which enabled relatively high storage moduli to be achieved compared to experiments performed at 10% w/w solids (see Figure 7).

Inspecting the TEM images obtained during construction of the pseudo-phase diagram shown in Figure 5, the mean worm cross-sectional thickness clearly increases when targeting higher PHBA DPs (see Figure 5 and Figure S3). In principle, thicker worms should be less flexible and hence exhibit longer persistence lengths than relatively thin worms. If so, this should lead to a greater number of inter-worm contacts within the 3D percolating network, which should enhance the gel strength and reduce the critical gelation concentration (CGC).

Tube inversion experiments performed at 20 °C for PGMA_70_-PHBA_175_, PGMA_100_-PHBA_250_, and PGMA_130_-PHBA_325_ (GMA/HBA molar ratio = 2.5) worm dispersions suggest that the CGC is indeed lowered (from 16 to 14 to 12% w/w, respectively; see Figure S7) with increasing PHBA DP and hence worm cross-sectional diameters (which are 32, 36, and 57 nm for GA-cross-linked PGMA_70_-PHBA_150_, PGMA_100_-PHBA_210_, and PGMA_130_-PHBA_270_ worms prepared at 37 °C, respectively; see Figure S6). These data are consistent with observations made by Lovett *et al.* for a PGMA_56_-PHPMA_155_ block copolymer worm gel.^61^

Finally, rheological studies of a 20% aqueous dispersion of PGMA_130_-PHBA_270_ nano-objects during a thermal cycle suggested a sphere-to-worm-to-sphere transition (Figure 8d). However, the storage modulus obtained at 23 °C is almost an order of magnitude higher during the cooling run compared to that observed during the initial heating run, which suggests significant hysteresis for this relatively concentrated dispersion (Figure 8d).

## Conclusions

HBA is evaluated as an alternative monomer to HPMA in the context of aqueous PISA syntheses. A kinetic study of the RAFT aqueous dispersion polymerization of HBA at 30 °C using a PGMA_100_ precursor was conducted while targeting a PHBA DP of 650. The reaction mixture was periodically sampled for analysis by ^1^H NMR spectroscopy, DMF GPC, and DLS. The ^1^H NMR data indicated that essentially full monomer conversion was achieved within 60 min. DMF GPC studies confirmed the linear evolution of *M*_n_ with monomer conversion. However, MWDs became significantly broader (*M*_w_/*M*_n_ > 1.40) above 65% conversion owing to the development of a high molecular weight shoulder arising from chain transfer to the acrylic backbone of the weakly hydrophobic PHBA chains. DLS studies indicated the formation of relatively small, well-defined nascent spheres after 7.5 min, which corresponds to the onset of micellar nucleation. Subsequently, the copolymer morphology evolved to produce worms and subsequently vesicles. To corroborate these tentative morphology assignments, GA was employed to cross-link the PHBA chains and hence enable TEM analysis. TEM images recorded for GA-cross-linked PGMA_100_-PHBA_215-650_ nano-objects extracted during DLS studies nano-objects confirmed the progressive evolution from spheres to worms to vesicles during the HBA polymerization.

A series of PGMA_*x*_-PHBA_*y*_ diblock copolymers were prepared for *x* = 57–300 and *y* = 100–1580, and the morphology of the resulting nano-objects was assigned by visual inspection, DLS, and TEM studies. This systematic approach allowed the construction of a pseudo-phase diagram that enabled the reproducible synthesis of pure spheres, worms, or vesicles. Interestingly, the upper limit PGMA stabilizer DP for which pure worms and vesicles could be accessed proved to be significantly higher for the RAFT aqueous dispersion polymerization of HBA compared to that of HPMA. This was attributed to the highly mobile nature of the more weakly hydrophobic PHBA block. The synthesis of copolymer spheres, worms, and vesicles with higher overall DPs is desirable because the relative amount of the RAFT agent is correspondingly reduced, which enables the production of cheaper, less malodorous copolymers with minimal color. Moreover, it also provides access to thicker worms and vesicles with thicker membranes. The former should be useful for the further examination of percolation theory to account for the formation of 3D worm gel networks,^61^ while the latter is expected to provide a more effective barrier against the diffusion of small molecules.^115^

Finally, temperature-dependent rheological studies conducted on a 10% w/w aqueous dispersion of linear PGMA_100_-PHBA_325_ nano-objects between 2 and 60 °C indicated thermoreversible behavior, despite the relatively long PHBA block. However, significant hysteresis was unexpectedly observed during the cooling cycle. Rheological studies of 20% w/w aqueous dispersions comprising PGMA_70_-PHBA_150_, PGMA_100_-PHBA_210_, and PGMA_130_-PHBA_270_ indicated that a reversible sphere-to-worm transition occurred at essentially the same CGT of 30–32 °C. Moreover, increasing the PHBA content of the worms formed at 37 °C provides a convenient means of tuning the gel strength. In principle, such thermoreversible worm gels should be useful as next-generation cell storage media for biomedical applications. In this context, their significantly higher CGT (compared to that observed for the prototypical thermoresponsive PGMA-PHPMA worm gels reported earlier^60,82^) should ensure that cells experience minimal thermal shock when inducing degelation, which is an essential step for cell harvesting.

## Supporting Information Available

Experimental details; DMF GPC curves recorded for aliquots taken during the synthesis of the PGMA_100_-PHBA_650_ diblock copolymer; GPC data recorded using a refractive index (RI) and a UV detector of the PGMA_100_-PHBA_650_ diblock copolymer; pseudo-phase diagram constructed for PGMA_*x*_-PHBA_*y*_ diblock copolymer nano-objects after GA cross-linking; TEM images recorded for PGMA_*x*_-PHBA_*y*_ diblock copolymer nano-objects after GA cross-linking; summary of DMF GPC and DLS data obtained for a series of PGMA_70–130_-PHBA_*y*_ diblock copolymer nano-objects; variable temperature DLS studies of PGMA_100_-PHBA_325_ nano-objects; TEM images recorded for PGMA_70_-PHBA_150_, PGMA_100_-PHBA_210_, and PGMA_130_-PHBA_270_ worms cross-linked at 37 °C; and digital images recorded for tube inversion experiments (PDF)
