# Supplementary material for: Reversible Addition-Fragmentation Chain Transfer Aqueous Dispersion Polymerization of 4-Hydroxybutyl Acrylate Produces Highly Thermoresponsive Diblock Copolymer Nano-Objects
Source: Macromolecules. 2022 Jan 19;55(3):788–98. doi: 10.1021/acs.macromol.1c02431 (PMC9007527; doi:10.1021/acs.macromol.1c02431)
Supplement: Supplementary file 1 — ma1c02431_si_001.pdf [file ma1c02431_si_001.pdf]

Supporting Information for:

Synthesis of Thermoresponsive Diblock Copolymer Nano-Objects by RAFT Aqueous Dispersion Polymerisation

Juliana M. Cumming, Oliver J. Deane* and Steven P. Armes*

Experimental section………………………………………………………………….. 1

Figure S1. DMF GPC curves recorded for aliquots taken during the synthesis of $PGMA_{100}$-$PHBA_{650}$ diblock copolymer……………………………………………………… 5

Figure S2. GPC data recorded using a refractive index (RI) and a UV detector in series of $PGMA_{100}$-$PHBA_{650}$ diblock copolymer……………………………………….................. 5

Figure S3. Pseudo-phase diagram constructed for all of the $PGMA_x$-$PHBA_y$ nano-objects synthesised in the present study…………………………………………………… 6

Figure S4. Representative TEM images recorded for $PGMA_x$-$PHBA_y$ diblock copolymer nano-objects after GA crosslinking…………………………………………………………….. 7

Table S1. Summary of DMF GPC and DLS data obtained for a series of $PGMA_{70\text{-}130}$-$PHBA_y$ diblock copolymer nano-objects…………………………………………………….. 8

Figure S5. Variable temperature DLS studies performed on a dispersion of $PGMA_{100}$-$PHBA_{325}$ nano-objects…………………………………………………………………… 9

Figure S6. Representative TEM images recorded for $PGMA_{70}$-$PHBA_{150}$ $PGMA_{100}$-$PHBA_{210}$, and $PGMA_{130}$-$PHBA_{270}$ worms crosslinked at 37 °C using GA………………….. 9

Figure S7. Digital images recorded for tube inversion experiments of $PGMA_{70}$-$PHBA_{175}$, $PGMA_{100}$-$PHBA_{250}$ and $PGMA_{130}$-$PHBA_{325}$ diblock copolymer worms…………………… 10

Experimental Section

Materials

HBA monomer was kindly donated by BASF (Ludwigshafen, Germany) and was purified *via* extraction using *n*-hexane (twenty times) to remove diacrylate impurities. All chemicals used for HBA purification were purchased from Sigma-Aldrich (Dorset, UK) and were used as received. A $PGMA_{57}$ precursor was kindly donated by Dr N. J. W. Penfold. This precursor was prepared using methylated 4-cyano-4-(2-phenylethanesulfanylthiocarbonyl)sulfanylpentanoic acid (Me-PETTC), as reported

elsewhere.[1] Ascorbic acid (AsAc), potassium persulfate (KPS), glutaraldehyde (GA), and aqueous solutions of HCl and NaOH were purchased from Sigma-Aldrich (Dorset, UK). CD_3OD and D_2O were purchased from Goss Scientific Instruments Ltd. (Cheshire, UK). All other solvents were purchased from Fisher Scientific (Loughborough, UK) and were used as received. Deionized water was used for all experiments.

Methods

^{1}H NMR Spectroscopy. NMR spectra (CD_3OD; 64 scans averaged per spectrum) were recorded at 298 K using a 400 MHz Avance-400 spectrometer.

Gel Permeation Chromatography (GCP). 0.50% w/w copolymer solutions were prepared using ultrafiltered DMF. GPC measurements were performed using HPLC-grade DMF eluent containing 10 mM LiBr at 60 °C at a flow rate of 1.0 mL min^{-1}. A Varian 290-LC pump injection module was connected to two Polymer Laboratories PL gel 5 µm Mixed-C columns connected in series and a Varian 390-LC multi-detector suite (refractive index detector). Molecular weight data are expressed relative to sixteen near-monodisperse poly(methyl methacrylate) standards ranging from M_p = 645 g mol^{-1} to 2 480 000 g mol^{-1}.

Dynamic Light Scattering (DLS). DLS studies were conducted at 20 °C (unless stated otherwise) and equilibrated for 120 s using a Malvern Instruments Zetasizer Nano series instrument equipped with a 4 mW He–Ne laser (λ = 633 nm) with the scattered light being detected at a fixed angle of 173°. Data were averaged over three consecutive measurements, each comprising ten runs of thirty seconds duration. Aqueous copolymer dispersions were diluted to 0.10% w/w and then adjusted to pH 3 using the appropriate amount of 1 M HCl. Intensity-average hydrodynamic diameters were calculated *via* the Stokes-Einstein equation, which assumes monodisperse, non-interacting spheres. In the case of the worm morphology, only a *sphere-equivalent* diameter is reported which corresponds to neither the worm contour length nor the worm width.

Transmission Electron Microscopy (TEM). Copolymer dispersions were diluted to 0.05% w/w solids using acidified deionized water (pH 3). Copper/palladium TEM grids (Agar Scientific, UK) were then coated in-house to produce thin films of amorphous carbon. These grids were then treated with a plasma glow discharge for 30 s to create

a hydrophilic surface. One 20 µL droplet of a 0.05% w/w aqueous copolymer dispersion was placed on a freshly-treated grid for 1 min and then blotted using filter paper to remove excess solution. To stain the deposited nanoparticles, a 10 µLdroplet of a 0.75% w/w aqueous solution of uranyl formate was placed on the sample-loaded grid via micropipet for 45 s and then carefully blotted to remove excess stain. Each grid was then dried using a vacuum hose. Imaging was performed using a Philips CM100 instrument operating at 100 kV and equipped with a Gatan 1k CCD camera.

Rheology. An AR-G2 rheometer equipped with a variable temperature Peltier plate and an aluminium cone (40 mm at 2°) was used for all rheological experiments. G′ and G″ were determined for as-synthesized 10-30% w/w aqueous copolymer dispersions as a function of temperature at an applied strain of 1.0% and an angular frequency of 1.0 rad s^{-1}. For each measurement, 30 s was allowed for thermal equilibration.

Synthesis of $PGMA_{57}$-$PHBA_x$ Diblock Copolymer Nanoparticles by RAFT Aqueous Dispersion Polymerization of HBA. $PGMA_{57}$-$PHBA_{200}$ nanoparticles were prepared via RAFT aqueous dispersion polymerization of HBA according to the following protocol. $PGMA_{57}$ precursor (0.1500 g, 15.8 µmol) was added to a glass vial with subsequent addition of HBA (0.4565 g, 3.1664 mmol), water (pH 3, 2.00 g, target solids concentration = 20% w/w) and KPS (0.0377 g, 140 µmol, [$PGMA_{57}$]/[KPS] molar ratio = 5.0). This vial was then charged with a magnetic flea, sealed with a rubber septum and purged with nitrogen for 30 min. The vial was then immersed in an oil bath set at 30 °C and the reaction mixture was stirred at 350 rpm. A solution of deoxygenated ascorbic acid (AsAc) (0.0245 g, 140 µmol; [KPS]/[AsAc] molar ratio = 1.0) and water (pH 3, 0.3724 g) was then added using a deoxygenated syringe and needle. The polymerization was allowed to proceed at 30 °C for 2 h before being quenched by exposing the reaction mixture to air while cooling in an ice bath. ^{1}H NMR spectroscopy studies confirmed that more than 99% HBA conversion was achieved while DMF GPC studies indicated an M_n of 42.7 kg mol^{-1} and an M_w/M_n of 1.32. A series of $PGMA_{57}$-$PHBA_x$ diblock copolymer nano-objects were prepared using a similar protocol by systematically varying the [HBA]/[$PGMA_{57}$] molar ratio. The final solids concentration was varied between 10 and 30% w/w by adjusting the amount of water added to the reaction mixture.

Synthesis of $PGMA_x$-$PHBA_y$ Diblock Copolymer Nanoparticles via RAFT Aqueous Dispersion Polymerization of HBA from a $PGMA_{57}$ Macro-CTA. A typical synthesis of $PGMA_{100}$-$PHBA_{300}$ nanoparticles at 20% w/w solids was conducted via RAFT aqueous dispersion polymerization of HBA using the following protocol. $PGMA_{57}$ precursor (95.0 mg, 10.0 µmol) was added to a glass vial with subsequent addition of GMA (0.0690 g, 430.7 µmol, target DP = 43, final overall PGMA DP = 100), water (0.1283 g, pH 2, target 40% w/w solids) and KPS (0.0126 g, 46.6 µmol, [$PGMA_{57}$]/[KPS] molar ratio = 10). After adding a magnetic flea, this vial was sealed and the aqueous reaction mixture was purged with nitrogen for 30 min before immersing the vial in an oil bath set at 30 °C. The reaction mixture was stirred at 350 rpm and a pre-mixed solution of deoxygenated AsAc (8.0 mg, 45.4 µmol, [KPS]/[AsAc] molar ratio = 1.0) in water (pH 3, 0.1000 g) was then added using a deoxygenated syringe and needle. The GMA polymerization was allowed to proceed at 30 °C for 2 h and then the vial was removed from the oil bath. ^{1}H NMR spectroscopy studies conducted on a 50 µL aliquot taken from the reaction mixture confirmed that 98% GMA conversion was achieved while DMF GPC analysis indicated an M_n of 20.7 kg mol^{-1} and an M_w/M_n of 1.23. HBA (0.4355 g, 3.021 mmol), water (pH 3, 1.1321 g) and KPS (0.0124 g, 0.0459 mmol, [$PGMA_{100}$]/[KPS] molar ratio = 10.0) were then added to the glass vial, which was sealed and purged with nitrogen gas for 30 min before being immersed in the same oil bath set at 30 °C. The reaction mixture was stirred at 350 rpm before adding a pre-mixed solution of deoxygenated AsAc (8.0 mg, 45.4 µmol; [KPS]/[AsAc] molar ratio = 1.0) and water (1.00 g) using a deoxygenated syringe. The HBA polymerization was allowed to proceed at 30 °C for 16 h. ^{1}H NMR spectroscopy studies indicated that more than 99% HBA conversion was achieved. DMF GPC studies indicated an M_n of 69.4 kg mol^{-1} and an M_w/M_n of 1.42. A series of $PGMA_x$-$PHBA_y$ diblock copolymer nano-objects were prepared using essentially the same protocol by systematically varying either the [GMA]/[$PGMA_{57}$] molar ratio or the [HBA]/[$PGMA_x$] molar ratio. The target solids concentration was varied by adjusting the amount of water added to the reaction mixture.

Covalent Stabilization of Diblock Copolymer Nano-objects Using Glutaraldehyde. A typical protocol used to crosslink $PGMA_{57}$–$PHBA_{100}$ spheres was as follows. An acidic aqueous solution of glutaraldehyde (GA; 50% w/w; 275 mg, 275 µmol, GA/HBA molar ratio = 0.66) was added to a 5.0% w/w acidic aqueous dispersion

of $PGMA_{57}$−$PHBA_{100}$ spheres (0.500 g; HBA residues = 417 µmol). This reaction mixture (pH 2-3) was stirred at 25 °C for 16 h. Then an aliquot (0.01 g) was extracted, diluted with water (4.99 g; final target polymer concentration = 0.05% w/w) and stirred for 24 h prior to TEM analysis. Covalent stabilization of worms and vesicles was also performed via GA crosslinking at 5.0% w/w solids using a GA/HBA molar ratio of 0.66.

Supporting Figures S1-S6

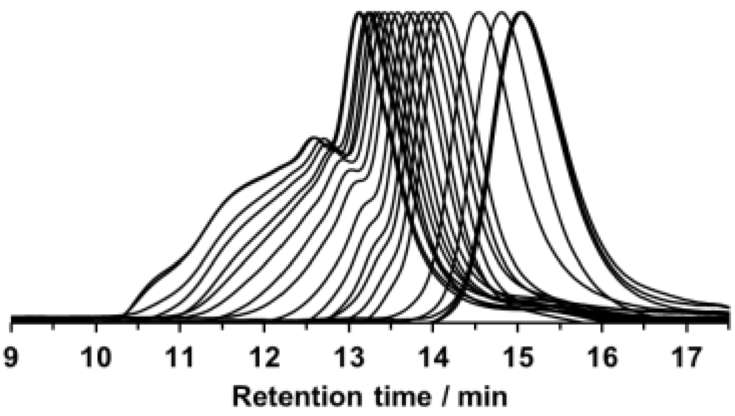

Figure S1. Representative DMF GPC curves recorded for aliquots taken during the synthesis of $PGMA_{100}$-$PHBA_{650}$ diblock copolymers prepared at 20% w/w solids *via* RAFT aqueous dispersion polymerization of HBA using a $PGMA_{100}$ precursor at 30 °C. Synthesis conditions: KPS/AsAc molar ratio = 1.0, $PGMA_{100}$/KPS molar ratio = 10.

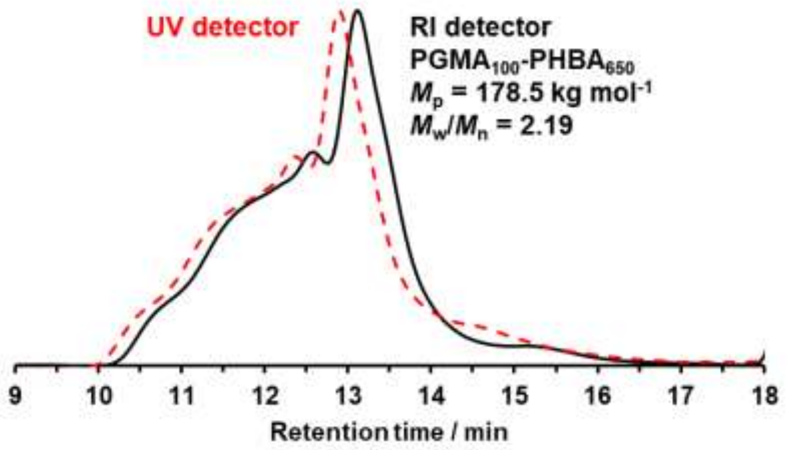

Figure S2. Representative DMF GPC curves obtained for a $PGMA_{100}$-$PHBA_{650}$ diblock copolymer prepared at 20% w/w solids *via* RAFT aqueous dispersion polymerization of HBA using a KPS/AsAc initiator at 30 °C. Data were recorded using a refractive index (RI) and a UV detector in series and are expressed relative to a series of near-monodisperse PMMA calibration standards.

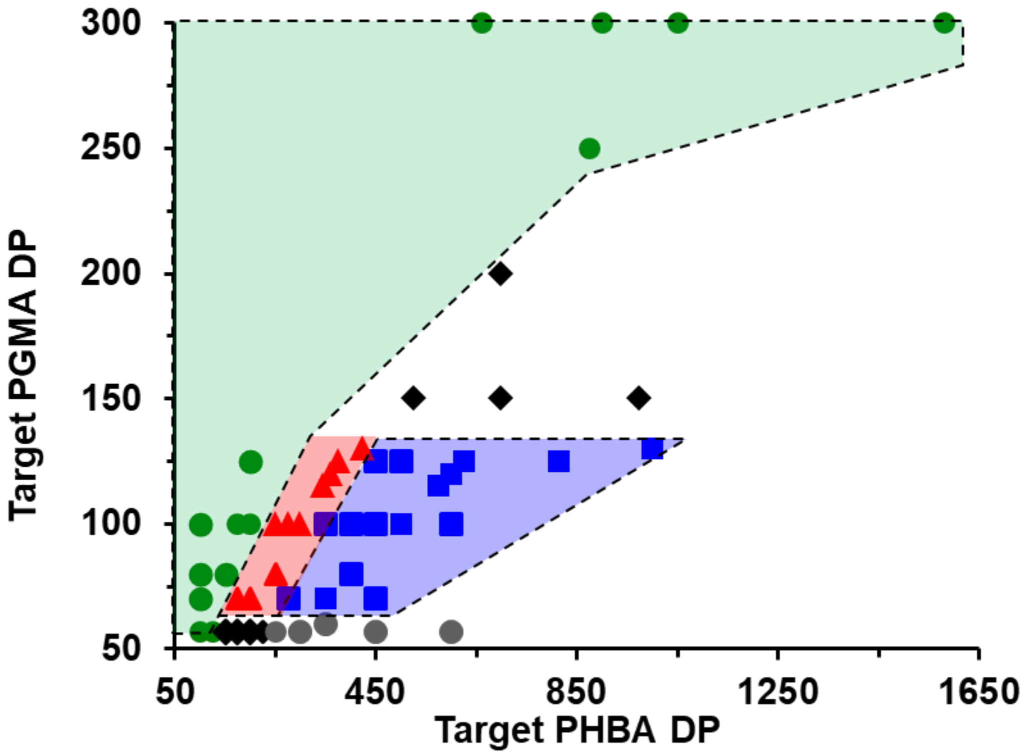

Figure S3. Pseudo-phase diagram constructed for all of the $PGMA_x$-$PHBA_y$ nano-objects synthesised in the present study. All syntheses involved the RAFT aqueous dispersion polymerization of HBA at 20% w/w solids at 30 °C. Each point represents the copolymer morphology assigned on the basis of DLS (non-crosslinked nano-objects) and TEM studies (after covalent stabilization at 20 °C using GA as a crosslinker). Green circles indicate spheres, red triangles indicate worms, blue squares indicate vesicles, black filled diamonds indicate mixed sphere/worms, and grey circles indicate macroscopic precipitation.

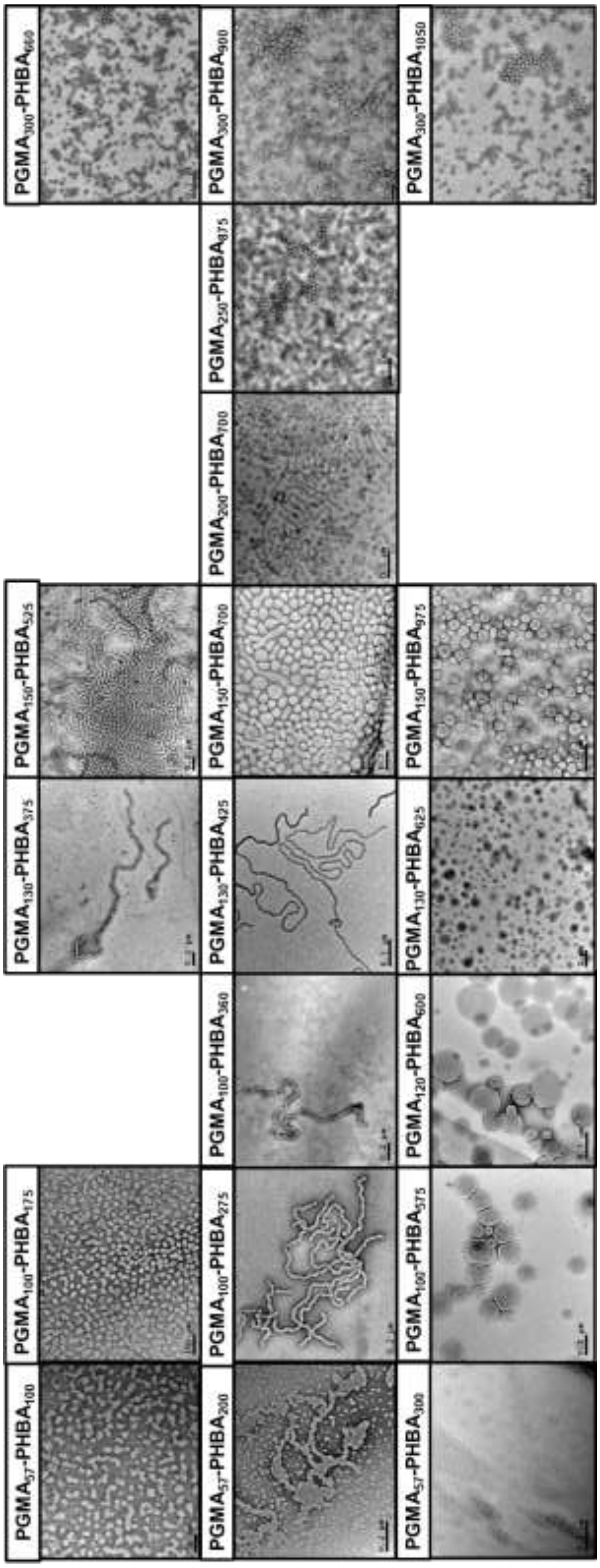

Figure S4. Representative TEM images recorded for $PGMA_x$-$PHBA_y$ diblock copolymer nano-objects after GA crosslinking of their PHBA chains at 20 °C. These images were used as part of the construction of the pseudo-phase diagram shown in the main manuscript (see Figure 5).

Table S1. Summary of DMF GPC and DLS data obtained for a series of $PGMA_{70-130}$-$PHBA_y$ diblock copolymer nano-objects prepared at 20% w/w solids *via* a one-pot protocol involving the RAFT aqueous solution polymerization of GMA immediately followed by the RAFT aqueous dispersion polymerization of HBA.

	GPC			DLS		
Polymer Composition	M_p / kg mol^{-1}	M_n / kg mol^{-1}	M_w/M_n	Diameter / nm	PDI	HBA Conversion
$PGMA_{70}$ precursor	19.2 ± 0.3	14.6 ± 0.3	1.25 ± 0.02	--	--	--
$PGMA_{70}$-$PHBA_{100}$	51.9	36.1	1.42	34	0.05	> 99%
$PGMA_{70}$-$PHBA_{175}$	68.4	42.5	1.67	1930	1.00	> 99%
$PGMA_{70}$-$PHBA_{200}$	72.7	56.8	1.39	5190	1.00	> 99%
$PGMA_{70}$-$PHBA_{275}$	102.2	78.2	2.15	643	0.29	> 99%
$PGMA_{80}$ precursor	22.2 ± 0.3	16.9 ± 0.5	1.25 ± 0.03	--	--	--
$PGMA_{80}$-$PHBA_{100}$	51.9	39.1	1.34	31	0.07	> 99%
$PGMA_{80}$-$PHBA_{150}$	63.8	45.2	1.46	41	0.03	> 99%
$PGMA_{80}$-$PHBA_{250}$	92.8	60.6	1.64	1138	0.40	> 99%
$PGMA_{80}$-$PHBA_{400}$	127.3	101	1.49	466	0.11	> 99%
$PGMA_{115}$ precursor	29.1 ± 0.3	20.7 ± 1.4	1.28 ± 0.07	--	--	--
$PGMA_{115}$-$PHBA_{345}$	111.5	83.9	1.43	152	0.24	> 99%
$PGMA_{115}$-$PHBA_{400}$	126.0	108.7	1.53	215	0.32	> 99%
$PGMA_{115}$-$PHBA_{575}$	165.6	140.6	1.49	386	0.14	> 99%
$PGMA_{120}$ precursor	32.0 ± 0.6	22.9 ± 0.7	1.31 ± 0.01	--	--	--
$PGMA_{120}$-$PHBA_{360}$	127.1	93.0	1.52	151	0.24	> 99%
$PGMA_{120}$-$PHBA_{420}$	142.5	100.2	1.70	209	0.24	> 99%
$PGMA_{120}$-$PHBA_{600}$	187.5	138.4	1.80	250	0.17	> 99%
$PGMA_{125}$ precursor	31.2 ± 0.6	21.8 ± 1.0	1.30 ± 0.04	--	--	--
$PGMA_{125}$-$PHBA_{200}$	88.0	61.7	1.57	33	0.09	> 99%
$PGMA_{125}$-$PHBA_{375}$	120.6	98.1	1.35	154	0.25	> 99%
$PGMA_{125}$-$PHBA_{450}$	175.2	180.9	2.60	212	175.2	> 99%
$PGMA_{125}$-$PHBA_{625}$	184.2	134.4	1.79	226	184.2	> 99%
$PGMA_{130}$ precursor	32.6	23.9	1.27	--	--	--
$PGMA_{130}$-$PHBA_{625}$	134.0	109.8	1.45	168	0.26	> 99%

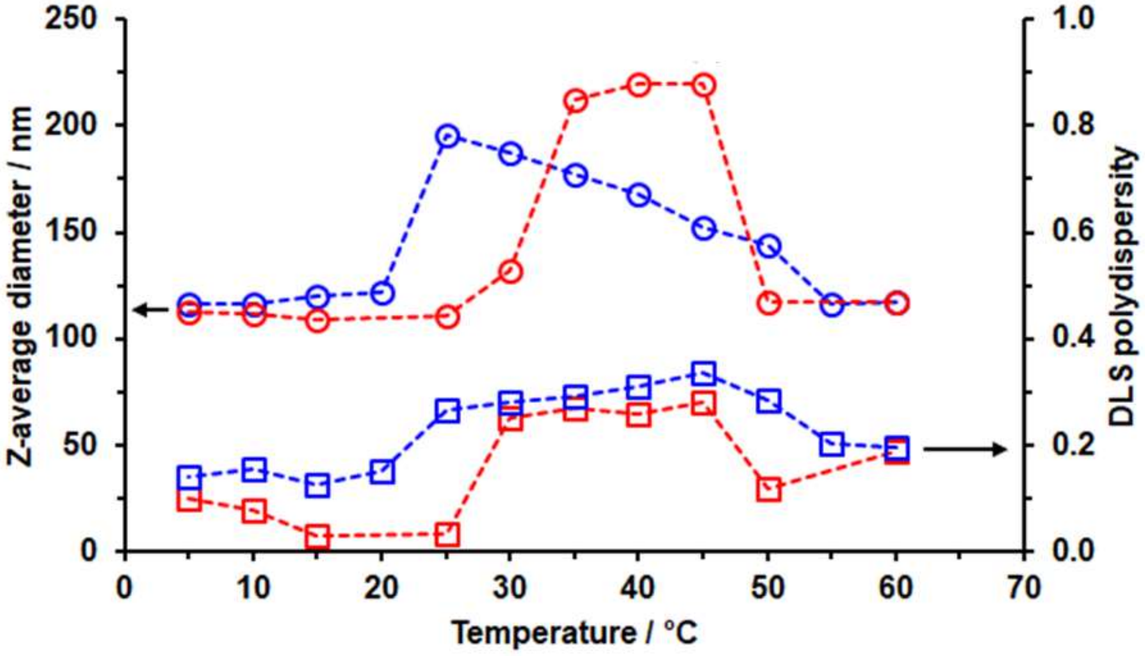

Figure S5. Variable temperature DLS studies performed on a 0.1% w/w aqueous dispersion of $PGMA_{100}$-$PHBA_{325}$ nano-objects. [z-average diameter = circles, DLS polydispersity = squares; heating = red data sets, cooling = blue data sets].

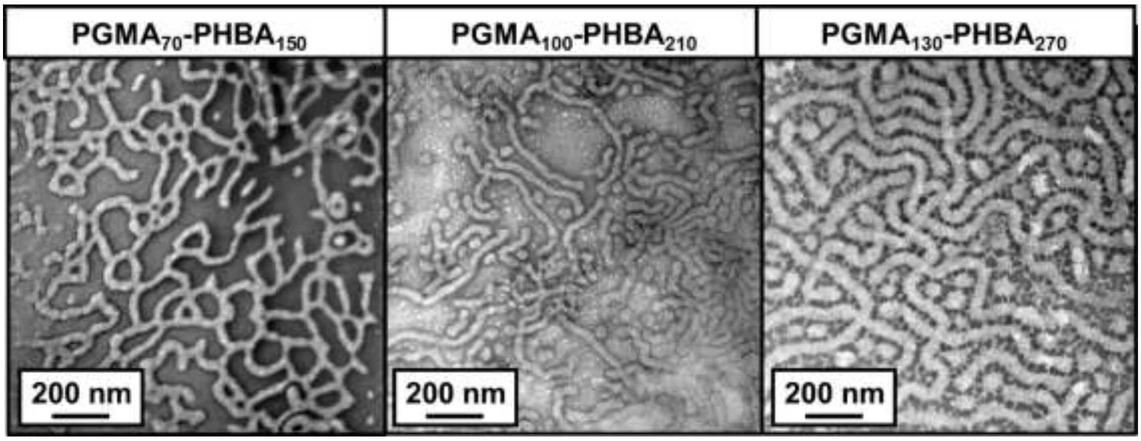

Figure S6. Representative TEM images recorded for $PGMA_{70}$-$PHBA_{150}$ (number-average worm diameter = 32 nm), $PGMA_{100}$-$PHBA_{210}$ (number-average worm diameter = 36 nm), and $PGMA_{130}$-$PHBA_{270}$ (number-average worm diameter = 57 nm) worms crosslinked at 37 °C using glutaraldehyde (GA).

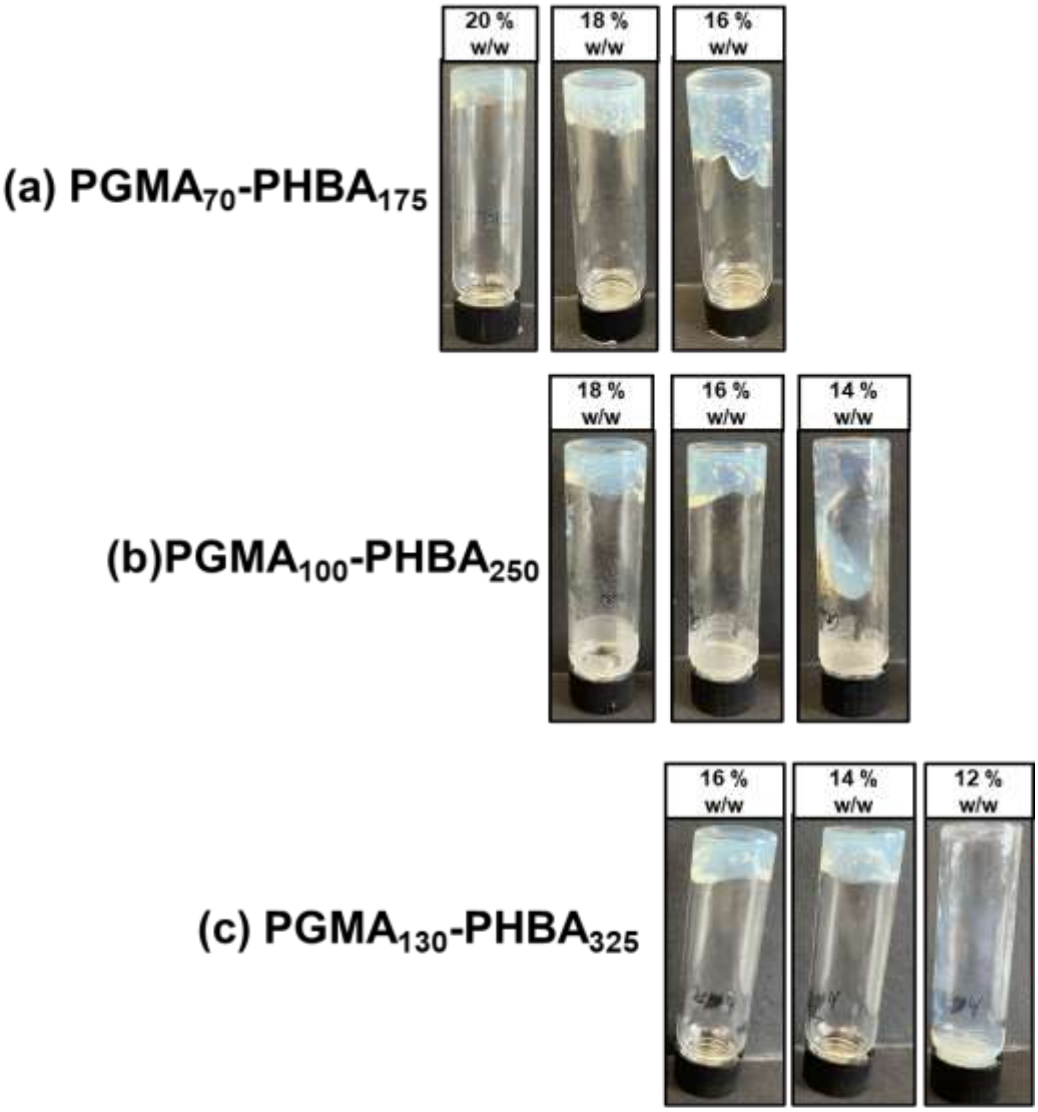

Figure S7. Digital images recorded for tube inversion experiments performed at 20 °C for various aqueous dispersions of $PGMA_{70}$-$PHBA_{175}$, $PGMA_{100}$-$PHBA_{250}$ and $PGMA_{130}$-$PHBA_{325}$ diblock copolymer worms to identify the critical gelation concentration (CGC).
